# E4orf1 improves adipose tissue-specific metabolic risk factors and indicators of cognition function in a mouse model of Alzheimer’s disease

**DOI:** 10.1038/s41387-023-00242-6

**Published:** 2023-08-12

**Authors:** Md Shahjalal Hossain Khan, Marleigh Hefner, Arubala Reddy, Nikhil V. Dhurandhar, Vijay Hegde

**Affiliations:** 1 Obesity and Metabolic Health Laboratory, Department of Nutritional Sciences, Texas Tech University, Lubbock, TX 79409 USA; 2 Neurosignaling Laboratory, Pennington Biomedical Research Center, Baton Rouge, LA USA

**Keywords:** Metabolic syndrome, Mouse

## Abstract

**Objective:**

Obesity, impaired glycemic control, and hepatic steatosis often coexist and are risk factors for developing dementia, and Alzheimer’s disease (AD). We hypothesized that a therapeutic agent that improves glycemic control and steatosis may attenuate obesity-associated progression of dementia. We previously identified that adenoviral protein E4orf1 improves glycemic control and reduces hepatic steatosis despite obesity in mice. Here, we determined if this metabolic improvement by E4orf1 will ameliorate cognitive decline in a transgenic mouse model of AD.

**Methods:**

Fourteen- to twenty-month-old APP/PS1/E4orf1 and APP/PS1 (control) mice were fed a high-fat diet. Cognition was determined by Morris Water Maze (MWM). Systemic glycemic control and metabolic signaling changes in adipose tissue, liver, and brain were determined.

**Results:**

Compared to control, E4orf1 expression significantly improved glucose clearance, reduced endogenous insulin requirement and lowered body-fat, enhanced glucose and lipid metabolism in adipose tissue, and reduced de novo lipogenesis in the liver. In the brain, E4orf1 mice displayed significantly greater expression of genes involved in neurogenesis and amyloid-beta degradation and performed better in MWM testing.

**Conclusion:**

This study opens-up the possibility of addressing glycemic control and steatosis for attenuating obesity-related cognitive decline. It also underscores the potential of E4orf1 for the purpose, which needs further investigations.

## Introduction

Obesity is a risk factor for the development of various metabolic diseases, such as impaired glycemic control and hepatic steatosis [1]. On the other hand, increasing body mass index (BMI) is correlated with an increased risk of developing dementia, including Alzheimer’s disease (AD) [2]. AD is a progressive neurodegenerative disease, with aging as an extremely significant risk factor [3]. Moreover, adipose tissue derived molecules, such as leptin and adiponectin, and their relationship with proinflammatory cytokines affect the progression of AD [4, 5]. Collectively, obesity, impaired glycemic control, and hepatic steatosis (liver fat accumulation) in association or independently are risk factors for neurodegeneration leading to AD [6] or other cognitive impairment and dementia [7]. If and how the obesity-related metabolic dysfunction affects AD remains unclear.

Interestingly, a common key factor in these pathological conditions is the impairment in insulin signaling. Fasting hyperinsulinemia is a widely used surrogate measure of insulin resistance and predicts type 2 diabetes (T2D) in various populations. Insulin concentrations are consistently higher in AD patients compared to controls [8], suggesting that obesity and hyperinsulinemia are common in the pathogenesis of AD. Further, obesity and hyperinsulinemia also cause accumulation of lipids in hepatic cells altering hepatic metabolic pathways involved in de novo lipogenesis, fatty acid uptake, fatty acid oxidation, and triacylglycerol export [9], which accelerates the progression of AD [10].

However, treatment strategies aimed at weight loss in individuals with midlife obesity have not shown reduction in incident AD, even though metabolic improvement due to weight loss were observed with these individuals [11]. Testing the role of anti-diabetic agents in attenuating AD onset or progression have also not been very successful [11–13]. Even though obesity and T2D can be somewhat controlled using available drugs, that may not be enough to treat AD, as currently there are no drugs approved to prevent or treat hepatic steatosis and its progression. In light of these studies, we hypothesized that reduction in adiposity, glycemic impairment, and liver fat accumulation will attenuate cognitive decline. If this is the case, further studies could be undertaken to identify the underlying mechanism. While this is conceptually straightforward, it is challenging to achieve reduction in adiposity long enough to chronically improve glycemic improvement or reduce hepatic steatosis [14]. Also, there is no effective medication for reducing hepatic steatosis, and addressing glycemic impairment alone with diabetes medications has not meaningfully impacted cognitive decline [15]. Hence, we asked if improving adiposity, glycemic impairment and hepatic steatosis simultaneously will help in reducing cognitive deterioration.

It appears that the E4orf1 protein originally isolated from human adenovirus type36 (Ad36) is a candidate to test our hypothesis. In vitro and in vivo evidence from our lab and others show that E4orf1 protein bypasses the proximal insulin signaling of insulin receptor (IR) and insulin receptor substrate-1 (IRS-1), and selectively enhances the Ras-mediated activation of phosphoinositide 3-kinase (PI3K) and phosphorylation of AKT to increase translocation of glucose transporter 4 (GLUT4) to the membrane, thereby promoting glucose uptake [16–19]. Consequentially, even in presence of insulin resistance, when insulin signaling is impaired, E4orf1 is able to promote glucose disposal and reduce response of endogenous insulin to glucose load [19].

Additionally, in presence of high-fat diet, E4orf1 significantly reduces liver fat accumulation in mice [20, 21]. To simultaneously test the effect of E4orf1-induced reduction in adiposity, glycemic impairment and liver fat on cognition, in this proof-of-concept study, we developed a transgenic mouse model that expressed E4orf1 in APP/PS1 mice—a model used for AD. For the current study, the triple transgenic mouse model (APP/PS1/E4orf1) were exposed to high-fat diet (HFD) induced obesity, and the changes in glycemic control, liver fat, adiposity, cognition were determined and relevant cell signaling was investigated to uncover potential underlying mechanism.

## Materials and methods

### Experimental design

A 20-week study design with two different diet compositions was used in this study. Transgenic APP/PS1 mice expressing APP and mutant PS1 from Jackson Laboratories [22] were bred with transgenic C57BL/6J mice expressing Ad36E4orf1 protein in the adipose tissue upon doxycycline induction [17]. Details for design and development of the Ad36E4orf1 transgenic mice has been previously described [17]. Genotyping details in supplemental files.

Mice expressing both APP/PS1 and E4orf1 transgene, APP/PS1/E4orf1 were used in the treatment groups and APP/PS1 transgene expressing mice were used as control. Transgenic expression of E4orf1 protein specific to the adipose tissue was confirmed in protein lysates from ingunal (subcutaneous) adipose depots, immunoblotted with E4orf1 antibody (Fig. S1). Mice were obtained from different breeding pairs. During experiments the mice were assigned numbers and the experimenter was blinded to their group assignment.

### Animal model

Fourteen- to twenty-month-old male and female mice, expressing E4orf1 only in the adipose tissue were grouped as APP/PS1/E4orf1 mice (*n* = 11) and non-transgenic APP/PS1 littermates (*n* = 7) as control group. All animals were housed in the same room ≤5 mice/cage on a 12‐h light/12‐h dark cycle. The experimental procedures were approved by the Texas Tech University Institutional Animal Care and Use Committee. Mice had access to ad-libitum diet and water. This study had two diet challenges; initially, mice were offered a high-fat-doxycycline diet (HFD-Doxy, 60% Kcal from fat, dox 600 ppm/kg) for 10 weeks followed by chow-doxycycline diet (chow-dox, 16%Kcal from fat, dox 600 ppm/Kg) for an additional 10 weeks. Mice were weighed weekly and body composition (fat mass and lean mass) measured using EchoMRI (EchoMRI LLC, Houston, TX) at the end of the study. Glucose tolerance test (GTT) was performed using oral gavage of glucose (2 g/Kg body weight) following fasting for 4 h. GTT was performed at 17–23 months of age. During GTT, serum glucose levels were measured by tail bleed at 0 and following glucose load at 15, 30, 60, and 120 min. Blood glucose level (mg/dl) was measured using AlphaTrak2 glucose meter. Blood was also collected at these time points in heparinized microvette (Sarstedt, cat. no. 16.444.100) for insulin measurement. Serum insulin level (ng/ml) was measured using ELISA kit (EMD Millipore, cat. no. EZRMI-13K). HOMA-IR value was calculated using equation HOMA-IR = Fasting blood glucose (mg/dl) × Fasting insulin (ng/ml) × 0.072. At termination of the study (18–24-month of age), mice were euthanized by CO_2_ asphyxiation and cervical dislocation. Trunk blood was collected in non-heparinized tubes and blood serum obtained after spinning at 14,000 rpm for 15 min at 4 °C and stored in −80 °C. Liver, adipose tissue depots (ingunal, epididymal, and retroperitoneal), skeletal muscle, and brain were collected during necropsy and flash frozen in liquid nitrogen or RNALater (Invitrogen, cat. no. AM7020), for protein and RNA analysis, respectively.

### Hemoglobin A1C (HbA1C) measurement

Glycosylated hemoglobin HbA1C concentration in blood was measured using a commercially available kit (Crystal Chem, cat. no. 80310) according to the manufacturer’s guidelines. Briefly, 5 μl of fresh whole blood was used. To 112 μl of protease buffer (1A), 25 μl of prepared blood lysate was added along with 48 μl of buffer 1B and incubated for 5 min at 37 °C. Following, which 70 μl of enzyme solution was added and absorbance was measured at 700 nm using a microplate reader following incubation for 3 min at 37 °C.

### Real-time quantitative PCR

Total RNA was extracted from the adipose tissue, liver, and brain tissue using RNeasy® Plus Universal Mini Kit (cat. no. 73404) as previously described [20]. Specific primers for each gene were designed using Sigma Aldrich Oligo Architect software, listed in Supplemental Table S1. The relative amount of all mRNAs was calculated using the 2^−ΔΔCT^ method. Details in supplemental files.

### Western blotting

Protein lysate was extracted from the inguinal adipose tissue depot, liver, and brain tissue by lysing in modified radioimmunoprecipitation assay buffer (RIPA buffer; Cell Signaling Technology Cat no: 9806) as previously described [20]. Tissue protein abundance from western blotting was analyzed in ImageJ (Image Processing and Analysis in Java) Version 1.51 software and estimated using the densitometry method. Details in supplemental files.

### Brain tissue and serum Aβ measurement using sandwich ELISA

Hippocampal, cortex, and serum Aβ40 and Aβ42 were estimated using commercially available solid-phase sandwich ELISA kit (IBL America, cat. no. 27718 and 27719, respectively). Hippocampal and cortex tissue samples were processed with RIPA lysis buffer with a 1X protease inhibitor (cat K1008; APExBio). Proteins were estimated and equalized using standardized BCA colorimetric assay. Protein (10–20 μg) from the hippocampus and cortex, and 2 µl of serum collected during sacrifice was used. Both Aβ40 and Aβ42 were estimated following the manufacturer’s guidelines and absorbance measured at 450 nm using a plate reader.

### Morris water maze (MWM) for spatial memory testing

The spatial learning and memory capabilities of the mice were evaluated with MWM test [23]. Detailed description is provided in supplemental file.

### Statistical analysis

The current study could be 80% powered at two-sided *α* = 0.05 having *n* = 4 mice in each group. To account for unintended losses during experiments, procedures, and assays, we included >*n* = 4 mice per group. All results are presented as mean ± standard error of the mean. Comparison between two groups was calculated using stringent Welch’s *t*-test assuming unequal variance in the values for each group. Specifically, we used Welch’s *t*-test for most variables because it performs similarly to Student’s *t*-test yet controls better for type 1 error rates when variance between groups and sample size per group is unequal [24].

## Results

### E4orf1 expression improves glycemic control in older APP/PS1 mice

**Fig. 1:**
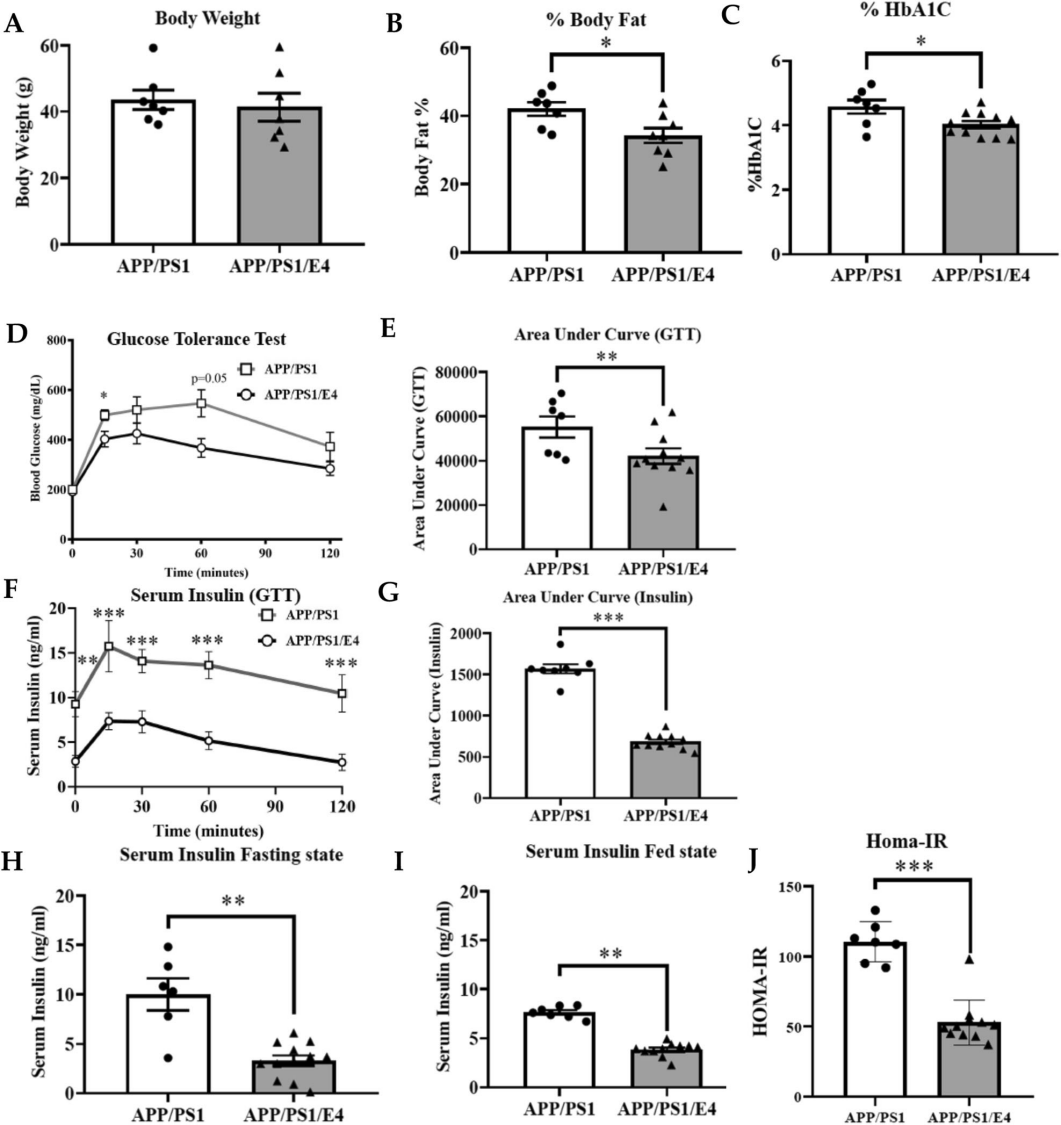
E4orf1 expression improves glycemic control in older APP/PS1 mice. E4orf1 expression in high fat fed APP/PS1 mice show no difference in average body weight over time (**A**), but significantly reduces % body fat (**B**), improves HbA1C (**C**), significantly lowers fasting (**D**) and fed (**E**) state serum insulin and improves insulin sensitivity as determined by HOMA-IR (**F**). Glycemic control was determined by glucose tolerance test (GTT) and corresponding insulin measurement. E4orf1 expressing APP/PS1 mice cleared glucose significantly faster (**G**, **H**) following a glucose challenge compared with control APP/PS1 mice requiring lower endogenous insulin (**I**, **J**). Welch’s *t* test: *p < 0.05, **p < 0.01.

Fourteen- to twenty-month-old APP/PS1/E4orf1 (*n* = 11; *n* = 5 males and *n* = 6 females) and APP/PS1 (*n* = 7; *n* = 3 males and *n* = 4 females) control mice were exposed to a 60% high-fat diet (HFD) supplemented with 600 mg/Kg doxycycline to induce E4orf1 expression. Following 10 weeks of HFD, the mice were switched to doxycycline supplemented rodent chow diet for an additional 10 weeks. At the end of the study, the mice were approximately 18–24 months in age and there was no difference in body weight between E4orf1 expressing and control APP/PS1 mice (Fig. 1A), but body fat percent was significantly lower (*p* < 0.05) in E4orf1 expressing mice (Fig. 1B) as determined by EchoMRI. To determine improvement in glycemic control over time (past 2–3 months), hemoglobin A1c (HbA1c) was measured. Fresh fasting blood collected from APP/PS1/E4orf1 mice shows significantly lower HbA1c values (*p* < 0.05) (Fig. 1C) compared with control APP/PS1 mice. To determine glycemic control in response to a glucose challenge, 17–23-month-old mice (~20 weeks of experimental feeding) were given an oral bolus of glucose (2.0 g/Kg) and glucose clearance measured via GTT. As seen in Fig. 1, APP/PS1 mice expressing E4orf1 were able to clear blood glucose significantly faster (*p* < 0.001; AUC) (Fig. 1D, E), requiring significantly lower endogenous insulin to clear the bolus of glucose compared with control APP/PS1 mice (Fig. 1F, G). Similarly, significantly lower insulin levels were observed under fasting (Fig. 1H) as well as fed state in APP/PS1/E4 mice (Fig. 1I), suggesting the requirement for less endogenous insulin to clear glucose in these mice compared with control mice. An indicator of insulin resistance, Homeostatic Model Assessment of Insulin Resistance (HOMA-IR), was determined based on the fasting glucose and fasting insulin levels. E4orf1 expressing mice show significantly lower HOMA-IR value (Fig. 1J; *p* < 0.001), which implies better insulin sensitivity compared with APP/PS1 control mice.

### E4orf1 expression in older APP/PS1 mice improves adipose tissue insulin and glucose molecular signaling

**Fig. 2:**
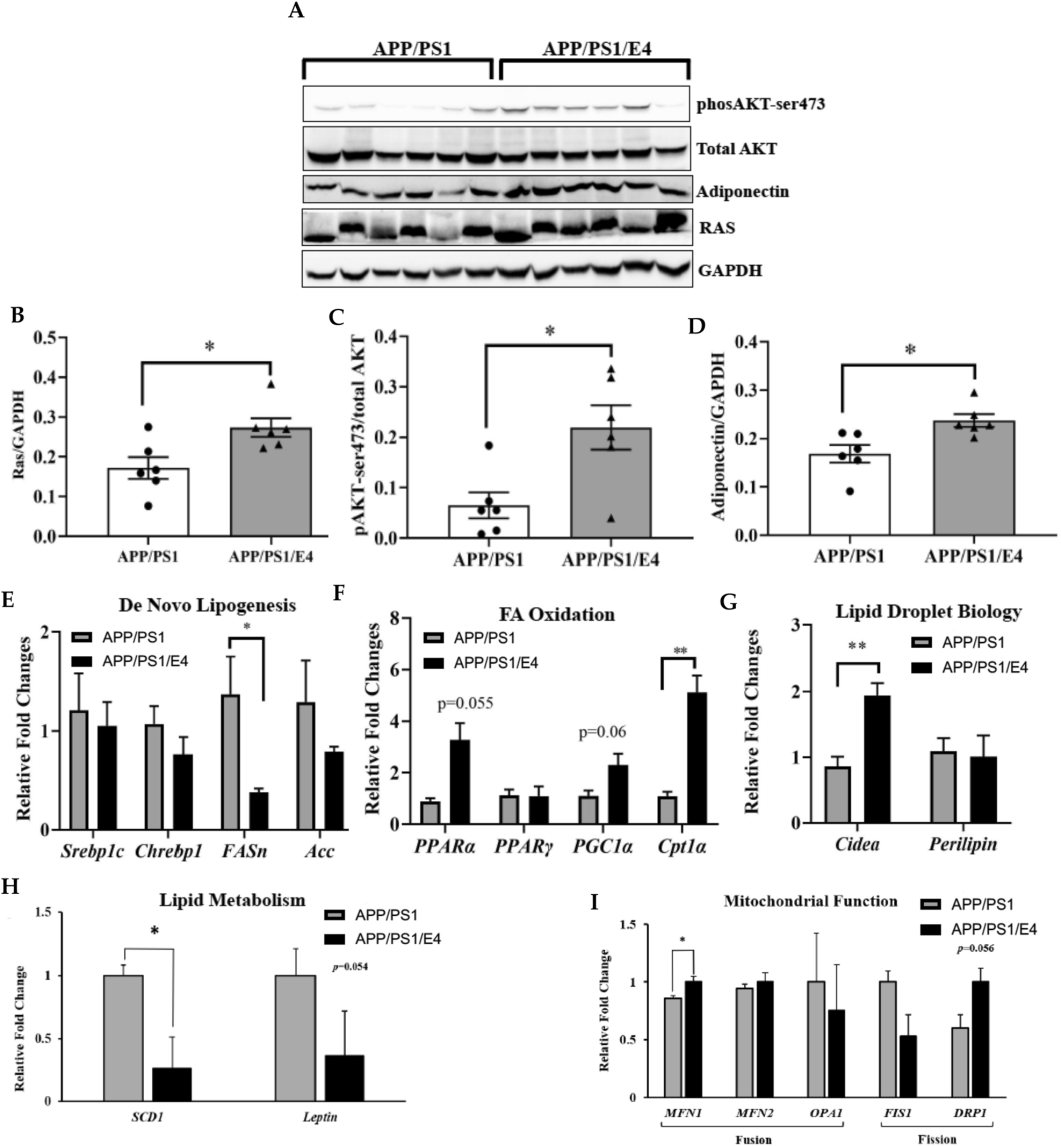
E4orf1 expression in older APP/PS1 mice improves adipose tissue insulin and glucose molecular signaling. APP/PS1 mice expressing E4orf1 improved Ras/PI3K/AKT insulin signaling in the ingunal adipose tissue by increasing protein expression of Ras (**A**, **B**) and phosphoAKT (**A**, **C**). Protein abundance of adiponectin was also significantly increased in E4orf1 mice compared with control mice (**A**, **D**). Protein expression was normalized to GAPDH or total protein (**A**) and densitometric analysis of the protein bands was plotted (**B**, **D**). Gene expression showed reduced de novo lipogenesis (**E**), increased fat oxidation (**F**), improved adipocyte lipid biology (**G**), lipid metabolism (**H**) and mitochondrial function (**I**). For gene expression APP/PS1 (*n* = 4) and APP/PS1/E4orf1 (*n* = 5). Welch’s *t* test: *p < 0.05, **p < 0.01.

The improvement in systemic glycemic control observed during GTT was further confirmed by molecular signaling changes in proteins and genes involved in adipose tissue insulin signaling, glucose uptake and lipid metabolism. In the inguinal (iWAT; subcutaneous) fat depot, E4orf1 expressing 18–24-month-old (following sacrifice) APP/PS1 mice show significantly increased expression of total Ras (Fig. 2A, B) and phosphoAKT (Fig. 2A, C) compared with control APP/PS1 mice indicating improved Ras/PI3K/AKT distal insulin signaling at the tissue level. E4orf1 expressing mice also show increased abundance of adiponectin (Fig. 2A, D), which regulates fatty acid and glucose catabolism, and is negatively correlated with type 2 diabetes (T2D). Further, E4orf1 expression in APP/PS1 mice significantly reduced expression of FASn, which is involved in de novo lipogenesis (Fig. 2E). For genes involved in fat oxidation, E4orf1 increased expression of Cpt1α (Fig. 2F), and increased gene expression of Cidea (Fig. 2G), involved in lipid biology and expanding adipose tissue cells to improve metabolic profile [25]. Compared with APP/PS1 control mice, APP/PS1/E4orf1 mice showed significantly lower expression of genes involved in lipid metabolism (SCD1) [26] (Fig. 2H) and increased expression of genes involved in mitochondrial fusion (MFN1) [27], but no difference for mitochondrial fission gene (Drp1) (Fig. 2I).

### E4orf1 expression in APP/PS1 mice protects against liver lipid accumulation

**Fig. 3:**
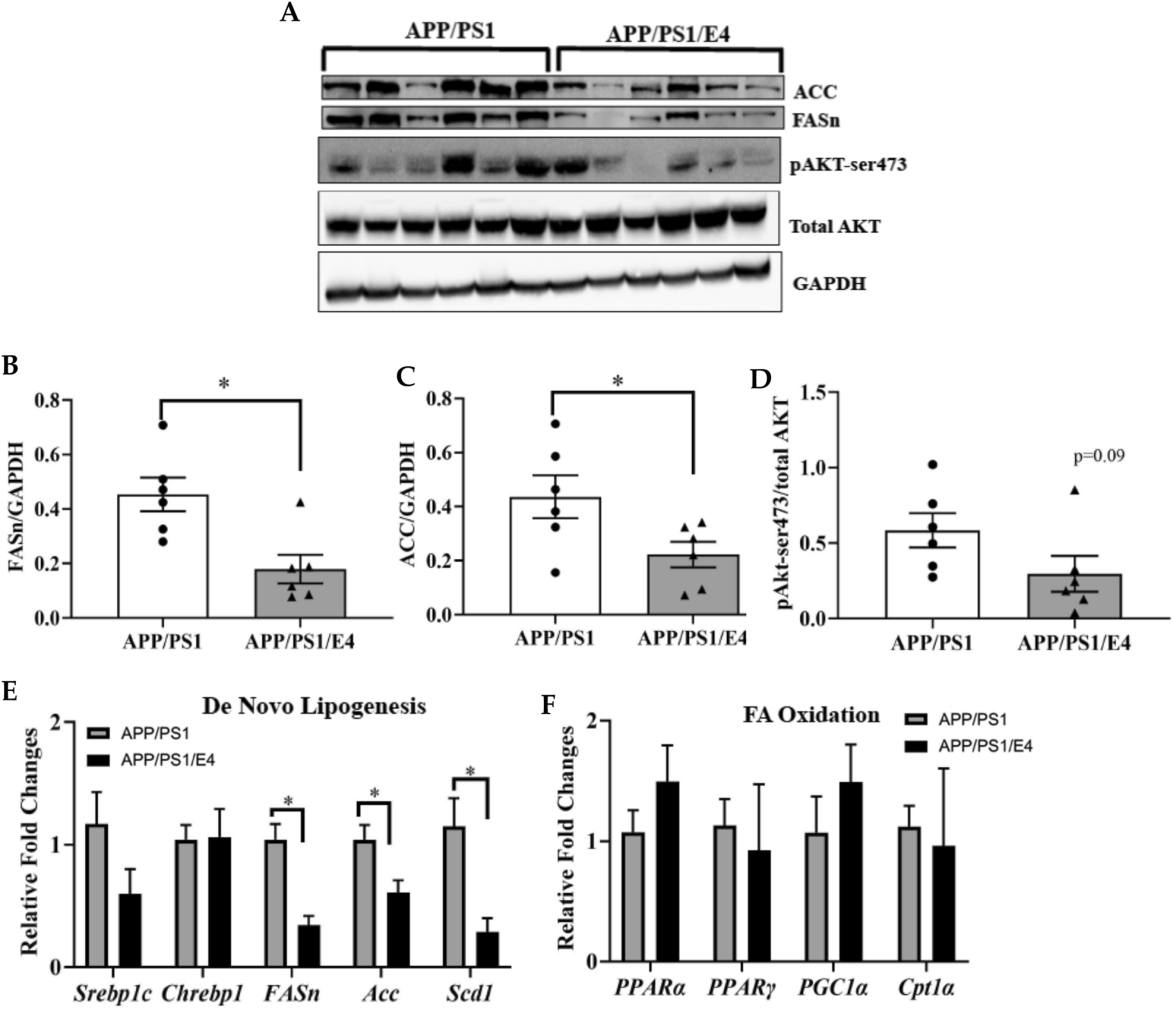
E4orf1 expression in APP/PS1 mice protects against liver lipid accumulation. Improved glycemic control with lower endogenous insulin secretion in E4orf1 expressing APP/PS1 mice prevents lipid accumulation in the liver as seen with significantly lower de novo lipogenesis, FASn (**A**, **B**) and ACC (**A**, **C**). Protein expression was normalized to GAPDH or total protein (**A**) and densitometric analysis of the protein bands was plotted (**B**, **D**). Hepatic gene expression also shows significantly lower lipogenesis related gene expression (FASn, ACC and Scd1) (**E**), while there was no significant difference in liver fat oxidation (**F**) in these mice. For gene expression APP/PS1 (*n* = 4) and APP/PS1/E4orf1 (*n* = 5). Welch’s *t* test: *p < 0.05, **p < 0.01.

Non-alcoholic fatty liver disease (NAFLD) has been suggested to influence neurological conditions associated with and promote AD in mice [10]. As seen in Fig. 3, E4orf1 expression in 18–24-month-old APP/PS1 mice shows significantly reduced expression for *FASn*, *ACC* and *Scd1* that are involved with de novo lipogenesis in the liver (Fig. 3A, C, E), protecting against hepatic lipid synthesis and subsequent accumulation. However, there was no significant difference in protein expression for phosphoAKT (Fig. 3A, D) and for genes involved with hepatic fat oxidation (Fig. 3F).

### E4orf1 expression mediated improvement in glycemic control prevents cognition decline in older APP/PS1 mice and ameliorates brain and serum amyloid beta levels

**Fig. 4:**
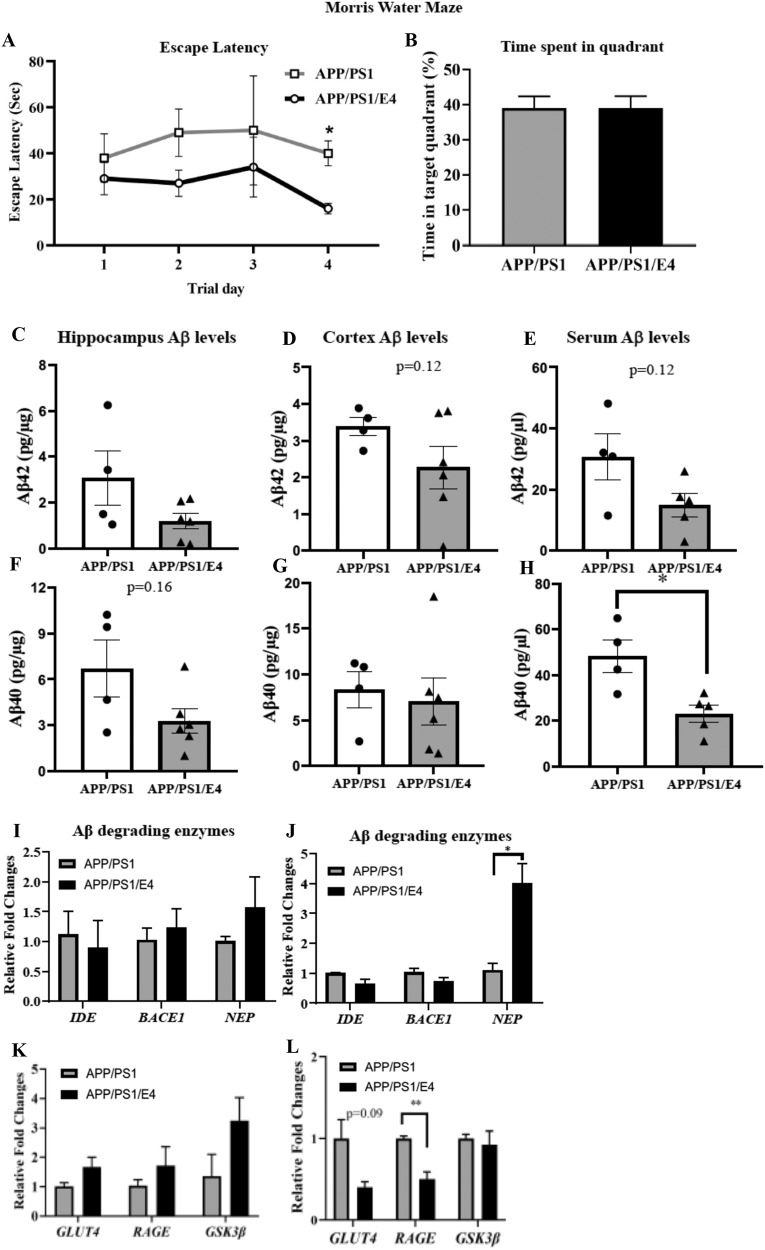
E4orf1 expression mediated improvement in glycemic control prevents cognition decline in older APP/PS1 mice and ameliorates brain and serum amyloid beta levels. To determine spatial learning and memory in the 17–23-month-old APP/PS1, morris water maze test was performed. Over the 4 trials done on different days, E4orf1 expressing APP/PS1 mice show faster escape latency by finding the platform, which is significantly improved on trial day 4 compared with control APP/PS1 mice (**A**). There was no significant difference in time spent in the quadrant when the platform was removed (**B**). In 18–24-month-old E4orf1 expressing APP/PS1 mice, there was no difference in Aβ expression levels in the brain hippocampal (**C**, **F**) and frontal cortex (**D**, **G**) regions. E4orf1 expressing mice show significant reduction in serum Aβ40 levels (**H**) but not Aβ42 (**E**). These mice also show significantly reduced expression of advanced glycation end-products (AGEs)-receptor (RAGE) (**L**) and increased expression of amyloid beta degrading enzyme neprilysin (NEP) (**J**) in the cortex, but not in hippocampus (**I**, **K**), suggesting reduced Aβ production and degradation. Welch’s *t* test: *p < 0.05.

We next wanted to test if improved peripheral glycemic control ameliorates cognitive decline in these mice. To determine spatial learning and memory, we performed the Morris water maze (MWM) test in 17–23-month-old mice (before sacrifice). Four trials with each lasting 60 s per day per mouse were performed for 2 consecutive days (Trial 1 and 2), then following a gap of 7 days, Trial 3 and 4 were performed. As seen in Fig. 4, the APP/PS1/E4orf1 mice were able to find the hidden platform faster compared with the control APP/PS1 mice, which significantly improved the escape latency (*p* < 0.007) on trial day 4 (Fig. 4A). However, we did not observe any difference with probe trial analysis for these mice when the platform was removed (Fig. 4B).

Significant amyloid deposition is a characteristic feature of all patients with AD. In 18–24-month-old E4orf1 expressing APP/PS1 mice, though not significant, there is a reduction in Aβ expression in the brain hippocampal (Fig. 4C, F) and cortical (Fig. 4D, G) regions. Additionally, there was significant reduction in Aβ40 levels in serum from these mice (Fig. 4H), suggesting lower circulating levels of Aβ in these mice. Further, these mice show significantly reduced expression of advanced glycation end-products (AGEs)-receptor (RAGE) in the cortex (Fig. 4L), but not in hippocampus (Fig. 4K) and also significantly increased expression of amyloid beta degrading enzyme neprilysin (NEP) in the cortex (Fig. 4J), but not in hippocampus (Fig. 4I).

### E4orf1 expression in APP/PS1 mice protects against brain mitochondrial dysfunction and neuronal health

**Fig. 5:**
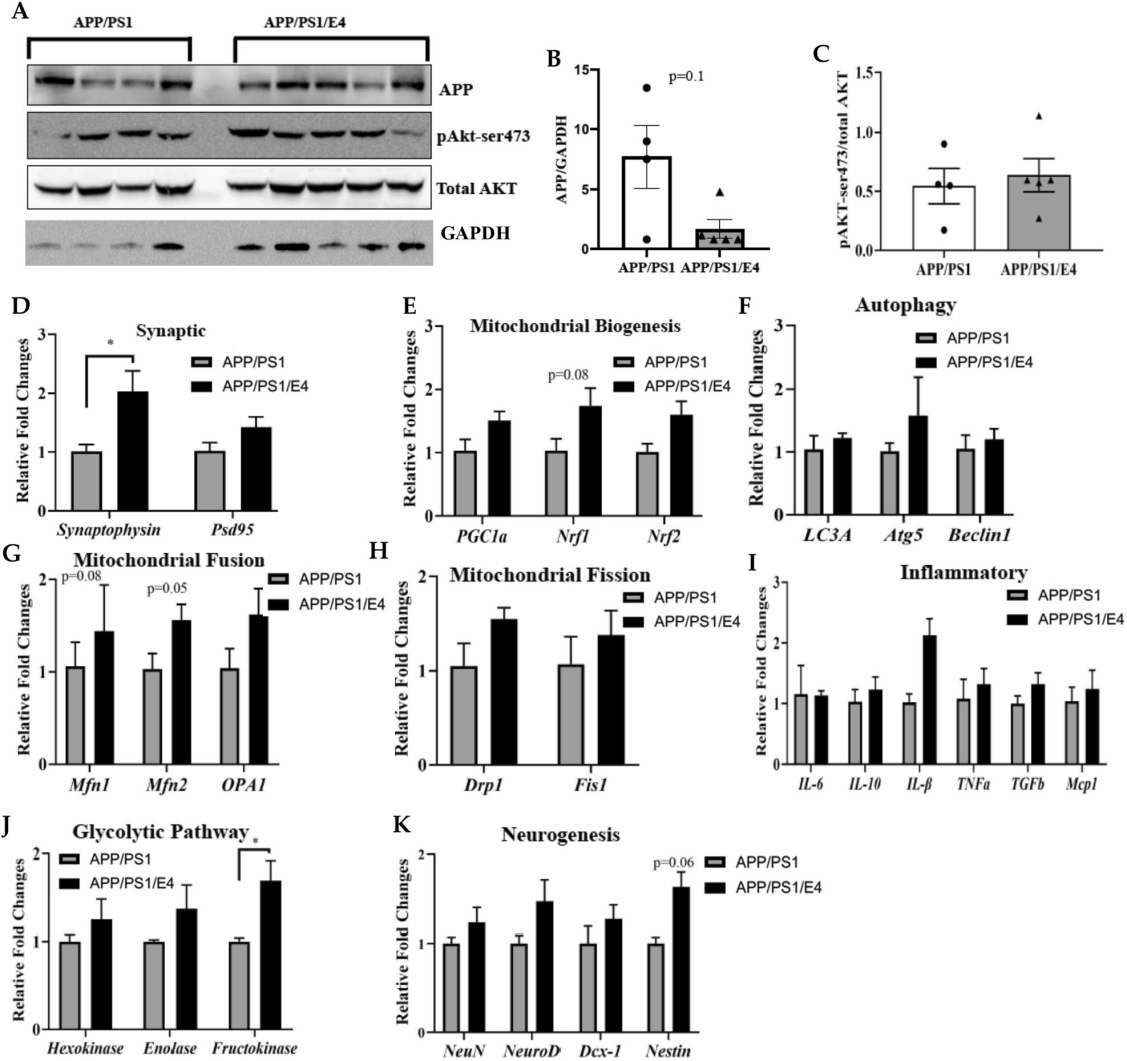
E4orf1 expression in APP/PS1 mice protects against mitochondrial dysfunction and neuronal health in the hippocampus. In the hippocampus, E4orf1 expressing APP/PS1 mice did not show any significant difference for protein expression of APP and phosphoAKT (**A**–**C**). Protein expression was normalized to GAPDH or total protein (**A**) and densitometric analysis of the protein bands was plotted (**B**, **C**). For gene expression, E4orf1 significantly increases expression of the presynaptic gene, synaptophysin (**D**), However, there was no difference in postsynaptic density protein, PSD95 (**D**). There was no significant difference in genes for mitochondrial biogenesis (**E**), autophagy (**F**), mitochondrial fusion (**G**) or fission (**H**), inflammation (**I**), and neurogenesis (**K**) between E4orf1 expressing and control APP/PS1 mice. Glycolytic gene fructokinase was significantly expressed in E4orf1 mice (**J**) suggesting better glucose metabolism. For gene expression APP/PS1 (*n* = 4) and APP/PS1/E4orf1 (*n* = 5). Welch’s *t* test: *p < 0.05.

**Fig. 6:**
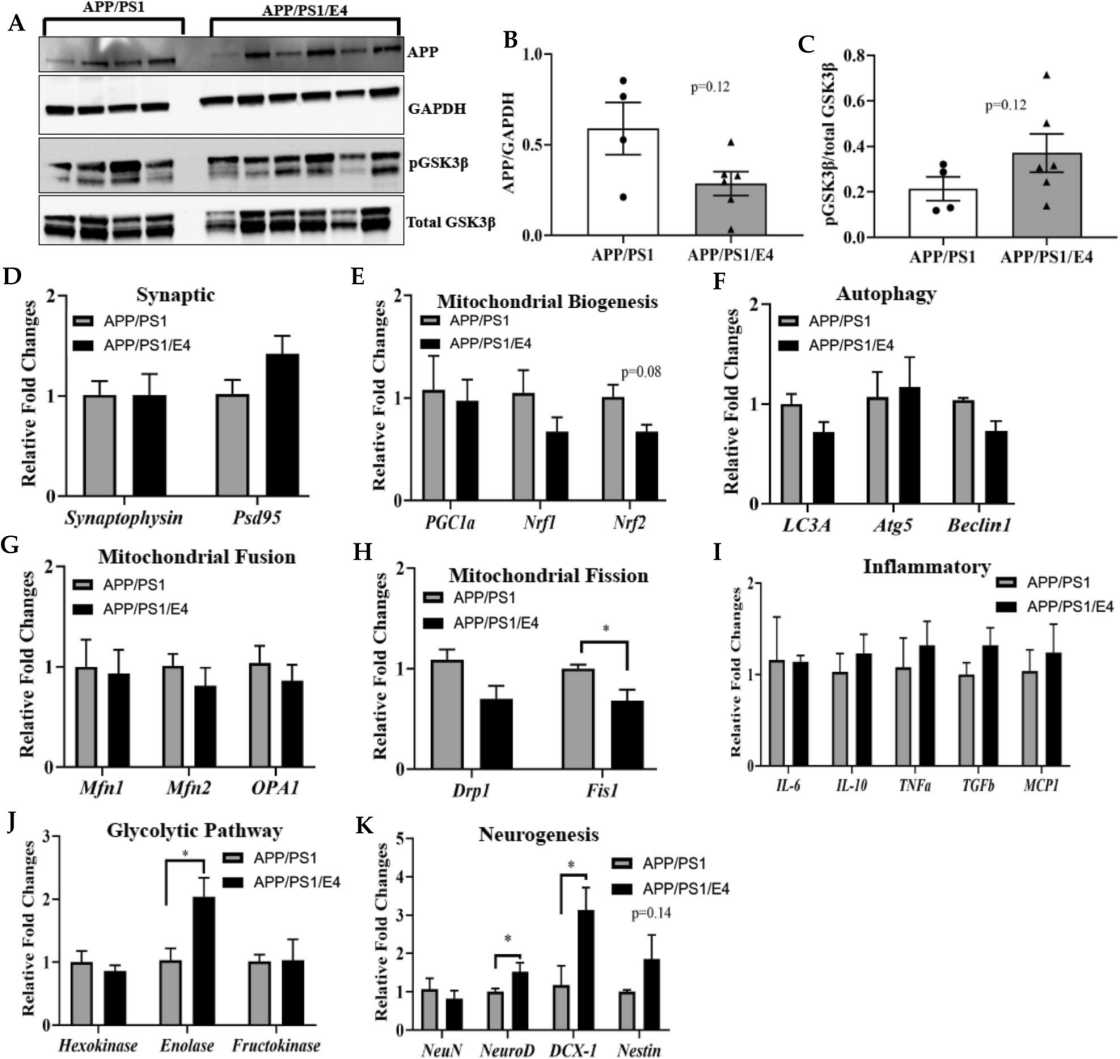
E4orf1 expression in APP/PS1 mice protects against mitochondrial dysfunction and neuronal health in the cortex. In the frontal cortex, there is no difference in protein expression of APP (**A**, **B**) and phosphoGSK3β (**A**, **C**). Protein expression was normalized to GAPDH or total protein (**A**) and densitometric analysis of the protein bands was plotted (**B**, **C**). There was no significant difference for genes involved in synapsis (**D**), mitochondrial biogenesis (**E**), autophagy (**F**), mitochondrial fusion (**G**), and inflammation (**I**), however, the expression of mitochondrial structure gene Fis1 was significantly reduced in E4orf1 expressing mice (**H**). Among the neurogenesis genes in the cortex, E4orf1 expressing mice have increased expression of neurogenic differentiation 1 (NeuroD) and one of its targets, double cortin (DCX-1) (**K**). Glycolytic gene Enolase is significantly expressed in E4orf1 expressing APP/PS1 mice (**J**) suggesting better glucose metabolism in the cortex compared with control APP/PS1 mice. For gene expression APP/PS1 (*n* = 4) and APP/PS1/E4orf1 (*n* = 5). Welch’s *t* test: *p < 0.05.

A large body of research has shown extensive mitochondrial abnormalities in the brain of AD patients and mitochondrial dysfunction has been established as an early and prominent feature of AD. Therefore, we determined the expression of mitochondrial genes in the hippocampus (Fig. 5) and frontal cortex (Fig. 6) of E4orf1 expressing and control APP/PS1 mice. In the hippocampus, E4orf1 did not show any significant difference for protein expression of APP and phosphoAKT (Fig. 5A, C). For gene expression, E4orf1 significantly increases expression of the presynaptic gene, synaptophysin (Fig. 5D), which is expressed in the neurons and has been implicated in AD pathology. However, there was no difference in postsynaptic density protein, PSD95 (Fig. 5D). Mitochondrial biogenesis plays an essential role in maintaining functional neuronal mitochondrial mass by compensating for damaged mitochondria and is thought to be impaired in AD, where the levels of nuclear respiratory factor 1 (Nrf1), nuclear respiratory factor 1 (Nrf2), and mitochondrial transcription factor A (TFAM) along with nuclear levels of peroxisome proliferator-activated receptor gamma, coactivator 1 alpha (PGC-1α) are reduced in the hippocampus. We did not observe any significant difference in mitochondrial biogenesis genes Nrf1, Nrf2, and PGC-1α (Fig. 5E) in E4orf1 expressing APP/PS1 mice compared with control APP/PS1 mice. Mitochondrial fusion protein Mfn2 showed significant increase in expression (Fig. 5G), but, we did not observe any significant difference in genes for autophagy (Fig. 5F), mitochondrial fission (Fig. 5H), inflammation (Fig. 5I), and neurogenesis (Fig. 5K) between E4orf1 expressing and control APP/PS1 mice. However, glycolytic gene fructokinase was significantly expressed in E4orf1 mice (Fig. 5J) suggesting better glucose metabolism. In the frontal cortex, similar to the hippocampus, there was no difference in expression of APP (Fig. 6A, B) and expression of phosphoGSK3β in E4orf1 expressing mice (Fig. 6A, C). Even though there was no significant difference for genes involved in synapsis (Fig. 6D), mitochondrial biogenesis (Fig. 6E), autophagy (Fig. 6F), and inflammation (Fig. 6I), the expression of mitochondrial structure fission gene Fis1 was significantly reduced in E4orf1 expressing mice (Fig. 6H), which is significantly increased in AD. Among the neurogenesis genes in the cortex, E4orf1 expressing mice show increased expression of neurogenic differentiation 1 (NeuroD) and one of its targets, double cortin (DCX-1) (Fig. 6K), which are important factors in mature neurons. Glycolytic gene Enolase is significantly overexpressed in E4orf1 expressing APP/PS1 mice (Fig. 6J) suggesting better glucose metabolism in the cortex compared with control APP/PS1 mice.

## Discussion

In this proof-of-concept study, we show that adenoviral protein, E4orf1 significantly reduced glycemic impairment, lowered body-fat, enhanced glucose and lipid metabolism in adipose tissue, and reduced de novo lipogenesis and steatosis in the liver. In the brain, E4orf1 mice displayed significantly greater expression of genes involved in neurogenesis and amyloid-beta degradation, thereby addressing obesity-related risk factors of cognitive impairment.

Despite the fact that obesity, impaired glycemic control and hepatic steatosis are metabolic conditions and AD is a central nervous system disease, an increasing number of epidemiological studies suggest that there is a significant comorbid association between obesity, impaired glycemic control, hepatic steatosis and AD [28]. However, the molecular mechanisms underlying this comorbidity and the influence of fat accumulation on the neurodegeneration process has not been well established.

In the current study, E4orf1 expression in APP/PS1 mice prevented body fat accumulation, improved systemic glycemic control with lower endogenous insulin requirement despite chronic high fat feeding. E4orf1 expression also significantly lowered HbA1c compared to control mice, which is an indicator of long-term maintenance of average blood glucose levels. It has been shown that elevated HbA1c is directly associated with increased risk of incident dementia and considered as a predictive biomarker of dementia and cognitive dysfunction [29]. Improved peripheral glycemic control appears to have prevented cognitive decline and improved spatial learning and memory as measured by MWM test. The E4orf1 expressing mice showed improved escape latency in finding the platform over the 4 trial days but there was no difference in probe analysis when the platform was removed. The lack of increased time spent in the platform quadrant could be due to insufficient training with the probe analysis and/or the age of the mice. At the molecular level, ingunal white adipose tissue (iWAT) showed improved insulin signaling by significantly increasing expression of Ras and pAKT in E4orf1 expressing APP/PS1 mice. More importantly, we observed increased expression of adiponectin exclusively expressed in the adipose tissue, which improves hyperglycemia by decreasing plasma glucose levels [30], and improves insulin sensitivity in mice. Furthermore, hypoadiponectinemia has been implicated in AD development [4]. In iWAT of APP/PS1 mice, E4orf1 expression improved lipid metabolism as determined by reduced expression of genes involved in de novo lipogenesis, increased expression of Cidea, which has a role in lipid droplet biology to prevent ectopic fatty acid accumulation in the liver and muscle [31] and increased fatty acid oxidation to prevent excess lipid accumulation in the adipose tissue. We also observed significantly reduced expression of stearoyl-CoA desaturase1 (SCD1) gene, known as an enzyme for lipid synthesis, which promotes lipid mobilization in iWAT [26]. Collectively, these observations highlight the improvement in systemic glycemic control and molecular improvement in lipid metabolism in E4orf1 expressing APP/PS1 mice compared with control APP/PS1 mice.

Hepatic lipid metabolism plays a role in the liver-brain axis and increased hepatic fat accumulation affects AD pathogenesis [10, 32]. Adipose tissue-specific E4orf1 expression in APP/PS1 mice, significantly reduced expression of protein and genes involved in hepatic de novo lipogenesis. These, observations suggest that prevention of hyperinsulinemia and improved adipose tissue lipid metabolism protects the liver from fat synthesis and accumulation in APP/PS1 mice expressing E4orf1.

Studies have shown extensive mitochondria abnormalities in the brain of AD patients [33] and mitochondrial dysfunction has been established as an early and prominent feature of AD [33, 34]. Therefore, we determined the expression of mitochondrial genes in the hippocampus and frontal cortex of E4orf1 expressing and control APP/PS1 mice. In the hippocampus, E4orf1 significantly increases expression of the presynaptic gene, synaptophysin, which is expressed in the neurons and has been implicated in AD pathology. Mitochondrial biogenesis is thought to be impaired in AD, where gene expressions are reduced in the hippocampus [35]. Even though there was no significant change in genes involved in mitochondrial biogenesis, glycolytic gene fructokinase was significantly expressed in E4orf1 mice suggesting better glucose metabolism in the hippocampus. In the frontal cortex, even though there was no significant difference for genes involved in synapsis, mitochondrial biogenesis, autophagy and inflammation, the expression of mitochondrial structure gene Fis1 is significantly reduced in E4orf1 expressing mice. Evaluation of AD brains have demonstrated significantly increased levels of fission factor Fis1 [36]. Among the neurogenesis genes in the cortex, E4orf1 expressing mice have increased expression of neurogenic differentiation 1 (NeuroD) and one of its targets, double cortin (DCX-1), which are important factors in mature neurogenesis. During development of the neocortex, the part of the cerebral cortex where higher cognitive functioning is thought to originate from, Neurod1 has been shown to promote terminal neuronal differentiation in progenitor cells. In adult humans, NEUROD1 expression appears to increase in cerebral cortex suggesting a protective mechanism by Neurod1 in the postnatal cerebral cortex [37]. On the other hand, Doublecortin (DCX) is required for normal migration of neurons into the cerebral cortex. Studies have shown that mutations in the human gene causes a disruption of cortical neuronal migration [38, 39]. Several studies have reported alterations of adult hippocampal neurogenesis in mouse models of AD [40, 41].

Advanced glycation end-products (AGEs) increase during normal aging, but their formation is promoted in glucose-rich environments, such as in hyperglycemia. Interestingly, elevated AGE levels are also observed in AD brains [42–44] and AGE-receptor (RAGE) was suggested as a possible receptor for Aβ [45] to mediate Aβ toxic mechanisms. Moreover, RAGEs have been shown to promote Aβ production, synaptic impairment, cognitive decline, and neurodegeneration [46]. E4orf1 expression in the cortex of APP/PS1 mice significantly reduces expression of RAGE and also significantly increases the expression of amyloid beta degrading enzyme neprilysin (NEP), suggesting reduced Aβ levels and increased degradation. We are cautious that even though gene expression may suggest functional changes, but firm conclusions cannot be drawn, therefore, further studies are warranted. Furthermore, we are unable to determine if the changes in the brain, and the changes in the glucose and fat metabolism are independent of each other or interdependent. Therefore, further studies are warranted to address this.

This study has some limitations as this was a cross sectional design and conducted in mice that were 18–24 months old at the end of the study, while most published behavioral studies in APP/PS1 mice are performed at a relatively younger age (around 8–15 months of age). This also is a strength of the study, where we were able to demonstrate that E4orf1 expression in APP/PS1 mice significantly improves spatial learning at this advanced age. The sample size and sex differences are also a limitation. Future study design should be a longitudinal study looking at peripheral changes, and their correlation with cognition over time to better establish the association between obesity, impaired glycemic control, hepatic steatosis and AD, and the effects of E4orf1. We are aware that findings from transgenic models cannot be used for clinical translation, therefore, we have developed nanoparticle mediated E4orf1 delivery method [47] and this will be used to finally outline pre-clinical strategies.

In conclusion, currently, there is no effective strategy to address obesity-related cognitive impairment and our study provides proof of concept that, it is possible to address obesity-related cognitive decline by reducing glycemic impairment and hepatic steatosis and identifies E4orf1 protein as a candidate therapeutic agent. Moreover, the fundamental understanding gained will also provide insight into the mechanism of peripheral metabolic impairments progressing to AD and help target therapies for prevention and better clinical management of AD.

### Supplementary information

Supplemental Material

## Data Availability

The datasets generated during and/or analyzed during the current study are available from the corresponding author on reasonable request.
