# Supplementary material for: E4orf1 improves adipose tissue-specific metabolic risk factors and indicators of cognition function in a mouse model of Alzheimer’s disease
Source: Nutr Diabetes. 2023 Aug 12;13:13. doi: 10.1038/s41387-023-00242-6 (PMC10423203; doi:10.1038/s41387-023-00242-6)
Supplement: Supplementary file 1 — Supplemental Material [file 41387_2023_242_MOESM1_ESM.pdf]

E4orf1 Improves Adipose Tissue-specific Metabolic Risk Factors and Indicators of Cognition Function in a Mouse Model of Alzheimer's Disease

Results

E4orf1 protein is expressed only in the adipose tissue of APP/PS1/E4 mice.

To confirm transgenic expression of E4orf1 protein specific to the adipose tissue, protein lysates from ingunal (subcutaneous), liver, hippocampus and cortex were immunoblotted with E4orf1 antibody. As seen in **Fig. S1**, western blot analysis showed E4orf1 protein expression only in the ingunal (iWAT) adipose tissue depot of APP/PS1/E4 and not in APP/PS1 mice. E4orf1 protein expression was not observed in the liver, hippocampus or cortex.

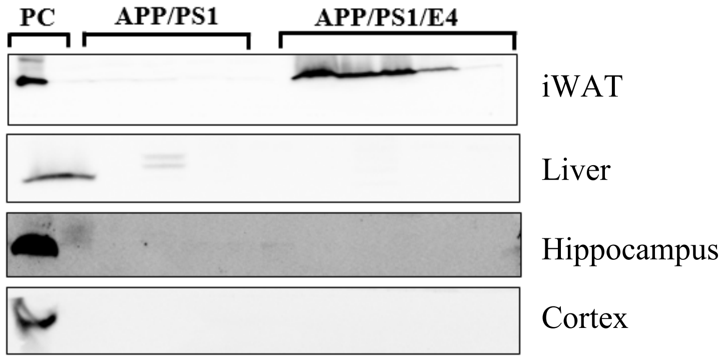

FIGURE S1: E4orf1 expression in APP/PS1/E4 mice adipose tissue. Protein lysates from ingunal (iWAT) adipose tissue shows E4orf1 expression only in APP/PS1/E4 mice but not in APP/PS1 mice. Protein lysates from liver, hippocampus and cortex of APP/PS1/E4 does not show E4orf1 expression confirming the adipose tissue specific expression of E4orf1. Protein lysates from previous positive mice was used as positive control (PC).

Methods

Genotyping for APP/PS1/E4orf1 transgene

Transgene-positive TRE-E4orf1 offspring were genotyped using PCR with the primer set: 5′-ATTGCTTCACCTCAGAGTGAGCG and 5′-AATCACTCTCTCCAGCAGCAGG (95°C: 2 minutes, 95°C: 15 seconds, 65°C: 45 seconds, 72°C: 20 seconds, Repeat steps 2-4 for 32 cycles, 72°C: 5 seconds, 4°C: Hold). Adiponectin-rtTA (APN-rtTA) mice were generated as previously described (1) and genotyped using PCR with the primer set: 5'-CTCTGGGAGAGGCGAGTATG and 5'-CAGGGTAGGCTGCTCAACTC. To confirm the presence of APP/PSEN1 genes, genotyping was performed by PCR using the following primers, APP 5'-AGGACTGACCACTCGACCAG and 5'-CGGGGGTCTAGTTCTGCAT and PSEN1 5'-AATAGAGAACGGCAGGAGCA and 5'-GCCATGAGGGCACTAATCAT. For APP/PSEN1 and APN-rtTA gentotyping the following touchdown PCR protocol was used 94°C: 2 minutes, 94°C: 20 seconds, 65°C: 15 seconds, 68°C: 35 seconds, Repeat steps 2-4 for 10 cycles, 94°C: 20 seconds, 60°C: 15 seconds, 72°C: 35 seconds, Repeat steps 6-8 for 28 cycles, 72°C: 5 minutes, 10°C: Hold.

Real-Time Quantitative PCR

Total RNA was extracted from the adipose tissue, liver and brain tissue using RNeasy® Plus Universal Mini Kit (cat. no. 73404). Tissue samples from each mouse was dissolved (~20 mg/900 µL) in QIAzol® reagent (cat. no. 79306) and homogenized with steel beads in Tissuelyser LT. Complementary DNA (cDNA) was synthesized using the Maxima H Minus First Strand Complementary DNA Synthesis Kit (Thermo Fisher Scientific, cat. no. K1681) with 1µg of RNA. The expression level of genes associated with adipose tissue, liver and brain tissue lipid metabolism, mitochondrial function and inflammation were determined by quantitative real-time polymerase chain reaction (qRT-PCR). Specific primers for each gene were designed using Sigma

Aldrich Oligo Architect software, listed in Supplemental Table S1. The RT-PCR reaction mix had a final volume of 20 µL; 50 or 25 ng of cDNA, 450 nM of the forward and reverse primers, and 10 µL of 1X SsoAdvanced™ Universal SYBR® Green Supermix (Bio-Rad Laboratories, cat. no. 172-5271). PCR reactions were carried out in 96-well plates using the Bio-Rad CFX RT-PCR detection system. All reactions were performed in duplicates. Mouse *B2m* and GAPDH genes were used as the reference.

Western Blotting

Protein lysate was extracted from the inguinal adipose tissue depot, liver and brain tissue by lysing in modified radioimmunoprecipitation assay buffer (RIPA buffer; 10X RIPA buffer from Cell Signaling (Cat no: 9806). 1X RIPA Buffer: 20 mM Tris-HCl (pH 7.5) 150 mM NaCl, 1 mM Na2 EDTA 1 mM EGTA 1% NP-40 1% sodium deoxycholate 2.5 mM sodium pyrophosphate 1 mM b-glycerophosphate 1 mM Na3 VO4 1 µg/ml leupeptin) as previously described. Protein extracts were separated using SDS-PAGE gel (Bio-Rad, Hercules, CA) and transferred on to a nitrocellulose membrane using Turbo transfer system (Bio-Rad, Hercules, CA). The nitrocellulose membrane was blocked using 10% non-fat milk in TBST (We utilized VWR's 20X liquid ultra-pure TBS Buffer (catalog number J640) and added a 1:100 ratio of Tween 20, using a 1X dilution for washing the western blot. We also used as a dilution buffer for the antibodies. The original compositions were as follows: When reconstituted with water, TBS 1X final concentrations are 137mM Sodium Chloride, 20mM Tris, 0.025% Tween-20. Supplied at pH 7.4 at +25°C) for an hour at room temperature, followed by immunoblotting with primary antibodies for E4orf1, phosphoAKT-ser473 (Cell Signaling #4060), RAS (Cell Signaling #3965), adiponectin (Cell Signaling #2789), Fatty acid synthase (FAS) (Cell Signaling #3180), phosphoGSK3α/β (Cell Signaling #9331) , total GSK3α/β (Cell Signaling #5676) or Glyceraldehyde 3-phosphate

dehydrogenase (Gapdh) (Cell Signaling #2118), at 1:1000-1:200 dilution. To visualize protein bands, the nitrocellulose membrane was treated with Clarity western ECL substrate (Biorad, cat. no. 170-5061) reagent following immunoblotting with appropriate HRP secondary antibody 1:2000 dilution. Tissue protein abundance from western blotting was analyzed in ImageJ (Image Processing and Analysis in Java) Version 1.51 software and estimated using the densitometry method. The intensity of protein bands was measured using blot densitometry in ImageJ, with background-subtracted ROI intensities. To select the band in image J, we choose rectangular selection in the area of interest and measure mean gray value as the measurement parameter for each selected band. We analyzed the density of individual band. To correct for local background noise, a nearby signal-free region on the blot was selected and subtracted from the average intensity of the protein band region. The resulting mean intensity measurement quantifies the amount of protein present in the band.

Morris water maze (MWM) for spatial memory testing

The spatial learning and memory capabilities of the mice were evaluated with MWM test (2). The test tank was 120 cm in diameter and 75 cm deep with visual cues placed around. The water tank was divided into four quadrants, north-west (NW), north-east (NE), south-west (SW), and south-east (SE). A circular opaque platform (36 cm in diameter) was located at the SW quadrant about 2 cm below the water level. A platform marker or 'flag' was mounted at the top of the platform to provide a visual cue to the position of the platform. The tank was filled with clear water, and the temperature was always maintained at 25-26 ^{o}C using an electric heater. The testing procedures were divided into three major phases: training phase, testing phase, and spatial probe test phase.

Phase 1 (training phase): During the training phase, the mice underwent three training sessions each day for two days. The mice were placed into the water from a randomly selected starting

position away from the fixed platform. This position was the same for all mice during that trial. The escape latency was set for 60 seconds, and after each trial, mice were placed on the platform for 20 seconds irrespective of if they reached or not. The time for escape latency was recorded using a stopwatch.

Phase 2 (testing phase): The testing session was performed for 2 consecutive days (Trial 1 and 2), and 2 additional days (Trial3 and 4) after a gap of 7 days. Once again, the platform was in the fixed position located at the SW quadrant. The starting position of the mice being placed in the tank was different at each trial. Each mouse had 3 trials per day for each trial, therefore each mouse went through 12 trials over the 4 days. The water was camouflaged to appear opaque using liquid tempera paint in the tank. The platform flag was removed during testing and the mice movement was video recorded.

Phase 1 (Training Phase)

Day	Trial 1	Trial 2	Trial 3
1	W	N	E
2	N	W	E

Phase 2 (Testing Phase)

Day	Trial 1	Trial 2	Trial 3
1	E	W	N
2	N	E	W
3	E	W	N
4	N	E	W

Phase 3 (spatial probe test): After, the mice underwent a one-time 60-second probe trial. The water was kept opaque, the platform removed, and the mice placed at a novel starting position. The

platform crossing times, percentage of time spent in the target quadrant, distance traveled in the target quadrant were recorded over a 60-second period.

Table S1: Primer sequences for genes used in RT-qPCR

Gene name		Primer Sequence (5'-3')
Fatp2	Forward	AGGCGACATCTACTTCAACA
	Reverse	CCATACACATTCACTTCTTCAACA
Fatp4	Forward	GACCAAGCCTACCTCACT
	Reverse	ACTGCCACATCTGCCATA
Fatp5	Forward	AGCCAGCCATCTTATCACAT
	Reverse	AAGCAGCCAAGGAATCCA
Lpl	Forward	CGCTCCATTCATCTCTTCATT
	Reverse	ACATCTTGCTGCTTCTCTTG
Srebp1c	Forward	GCTTCTCTTCTGCTTCTCTG
	Reverse	GGCTGTAGGATGGTGAGT
Chrebp	Forward	TTCCACAAGCATCCTGACT
	Reverse	AGAAGCGTGTTCACAAGTTG
Fasn	Forward	GTCGTCTATACCACTGCTTACT
	Reverse	ACACCACCTGAACCTGAG
Acc1	Forward	GCAGCAGTTACACCACATAC
	Reverse	TCCGCCATCTTCCACAATA
Scd-1	Forward	TGCCTCTTAGCCACTGAAT
	Reverse	ACTGTTGAGATGTGAGACTGT
Lipin1	Forward	GCCGTGTCATATCAGCAAT
	Reverse	ATCGCCAGAAGTAGAGGAG
Gpat1	Forward	CTATCCAGTAACGAGTCCAGAA
	Reverse	GGCGGTGAAGAGAATGTG
Gpat2	Forward	GTCTTCCTACTGCTACTGTCA
	Reverse	TGCTGTCTTCCTGTGTCA
Gpat3	Forward	GCTTGCTGATTATAGGAACTACAC
	Reverse	GCTTGTTATGGTAATGGATGGT
Gpat4	Forward	GGTGGAGAACAGCGAGTA
	Reverse	TCAGAAGGAAGGACAGAAGG
Dgat1	Forward	GATTGGTGGAATGCTGAGTC
	Reverse	GGCTTGTAGAAGTGTCTGATG
Dgat2	Forward	TCCAGAAGAAGTTCCAGAAGTAT
	Reverse	CAGGTGTCAGAGGAGAAGAG
L-fabp	Forward	GTCAAGGCAGTCGTCAAG
	Reverse	ATGGTATTGGTGATTGTGTCTC
Dbi	Forward	CCTCAAGACTCAGCCAACT
	Reverse	GTATTTACATCGCCCACAGTAG
Scp2	Forward	GCCAGGAGATGCTATGAGA
	Reverse	CCAGTGCTTCGTAAGTGATG

Pppar-α	Forward	TCGCTATCCAGGCAGAAG
	Reverse	ACAACAACAACAATAACCACAGA
Ppar-γ	Forward	CCACCAACTTCGGAATCAG
	Reverse	GCTCTTGTGAATGGAATGTCT
Cpt1α	Forward	CAAGCCAGACGAAGAACATC
	Reverse	TGACCATAGCCATCCAGATT
PGC1α	Forward	ACAATAACAACAACAACCATACCA
	Reverse	ATTCTGTCTCTTGCCTCTTCA
Drp1	Forward	ATGCCAGCAAGTCCACAGAA
	Reverse	TGTTCTCGGGCAGACAGTTT
Fis1	Forward	CAAAGAGGAACAGCGGGACT
	Reverse	ACAGCCCTCGCACATACTTT
Mfn1	Forward	GCAGACAGCACATGGAGAGA
	Reverse	GATCCGATTCCGAGCTTCCG
Mfn2	Forward	TGCACCGCCATATAGAGGAAG
	Reverse	TCTGCAGTGAACTGGCAATG
OPA1	Forward	ACCTTGCCAGTTTAGCTCCC
	Reverse	TTGGGACCTGCAGTGAAGAA
PGC1α	Forward	GCAGTCGCAACATGCTCAAG
	Reverse	GGGAACCCTTGGGGTCATTT
Nrf1	Forward	AGAAACGGAAACGGCCTCAT
	Reverse	CATCCAACGTGGCTCTGAGT
Nrf2	Forward	ATGGAGCAAGTTTGGCAGGA
	Reverse	GCTGGGAACAGCGGTAGTAT
Synaptophysin	Forward	CTGCGTTAAAGGGGGCACTA
	Reverse	ACAGCCACGGTGACAAAGAA
PSD95	Forward	CTTCATCCTTGCTGGGGGTC
	Reverse	TTGCGGAGGTCAACACCATT
LC3A	Forward	CCCATCGCTGACATCTATGAAC
	Reverse	AAGGTTTCTTGGGAGGCGTA
ATG5	Forward	TCCATCCAAGGATGCGGTTG
	Reverse	TCTGCATTTCGTTGATCACTTGAC
Beclin	Forward	ACCAGCGGGAGTATAGTGAGT
	Reverse	CAGCTGGATCTGGGCGTAG
Pink1	Forward	CCATCGGGATCTCAAGTCCG
	Reverse	GATCACTAGCCAGGGACAGC
G6pase	Forward	GGAAGGATGGAGGAAGGAAT
	Reverse	TCAGGTCAGCAATCACAGA
Pepck	Forward	GACATTGCCTGGATGAAGTT
	Reverse	CGTTGGTGAAGATGGTGTT
B2m	Forward	GAAGCCGAACATACTGAACTG
	Reverse	CTGAAGGACATATCTGACATCTCT
Nestin	Forward	AGATGAGCAGATGACAGTGA
	Reverse	AGTCTCCAGTGATTCTATGTTCT
NeuroD1	Forward	AGTTATTGCGTTGCCTTAGC

	Reverse	AGTGTTATGGGTCTGGTTTCT
NeuN	Forward	CTTCCACCGTCTCCTTCT
	Reverse	TTGTTGTTGTTGCTGTTGTTG
Dcx1	Forward	CATTAGCCATCACACCAAGG
	Reverse	TAGCCAATCTCACACCAGTT
IL-10	Forward	GTGATGCCCCAAGCTGAGA
	Reverse	CACGGCCTTGCTCTTGTTTT
TNFα	Forward	AAATGGGCTCCCTCTCATCAGTTC
	Reverse	TCTGCTTGGTGGTTTGCTACGAC
MCP1	Forward	AGAATCACCAGCAGCAAGTGTCC
	Reverse	TCCTGAACCCACTTCTGCTTGG
Tgfb	Forward	CAGCAACAATTCCTGGCGATA
	Reverse	AAGGCGAAAGCCCTCAATTT
IL-6	Forward	TCCTACCCCAACTTCCAATGCTC
	Reverse	TTGGATGGTCTTGGTCCTTAGCC

ABBREVIATIONS

Fatp2, solute carrier family 27 (fatty acid transporter), member 2; Fatp4, solute carrier family 27 (fatty acid transporter), member 4; Fatp5, solute carrier family 27 (fatty acid transporter), member 5; Lpl, lipoprotein lipase; Srebp1c, sterol regulatory element binding protein 1c; Chrebp, carbohydrate response element binding protein; Fasn, fatty acid synthase; Acc1, acyl CoA carboxylase 1; Scd1, stearoyl-Coenzyme A desaturase 1; Gpat1, glycerol-3-phosphate acyltransferase, mitochondrial; Gpat2, glycerol-3-phosphate acyltransferase 2, mitochondrial; Gpat3, glycerol-3-phosphate acyltransferase 3; Gpat4, glycerol-3-phosphate acyltransferase 4; Dgat1, diacylglycerol O-acyltransferase 1; Dgat2, diacylglycerol O-acyltransferase 2; L-fabp, liver fatty acid binding protein; Dbi, diazepam binding inhibitor; Scp2, sterol carrier protein 2, liver; Ppar-α, Peroxisome proliferator-activated receptor alpha; Ppar-γ, peroxisome proliferator activated receptor gamma; Pgc-1α, peroxisome proliferative activated receptor, gamma, coactivator 1 alpha; Cpt1a, carnitine palmitoyltransferase Ia; Hmgcs2, 3-hydroxy-3-methylglutaryl-CoA synthase 2; Cidea, cell death-inducing DNA fragmentation factor, alpha subunit-like effector A; G6pase, glucose 6-phosphatase; Pepck, phosphoenolpyruvate

carboxykinase; B2m, Beta-2-Microglobulin; IL-6, Interleukin 6, IL-10, Interleukin 10; IL-1b, Interleukin1b.

References

1. Wang QA, Tao C, Gupta RK, Scherer PE. Tracking adipogenesis during white adipose tissue development, expansion and regeneration. *Nat Med* 2013;**19:** 1338-1344.

2. Vorhees CV, Williams MT. Morris water maze: procedures for assessing spatial and related forms of learning and memory. *Nat Protoc* 2006;**1:** 848-858.
